# Predictors of Real-World Parents’ Acceptance to Vaccinate Their Children Against the COVID-19

**DOI:** 10.15388/Amed.2021.29.1.4

**Published:** 2022-07-26

**Authors:** Petros Galanis, Irene Vraka, Olga Siskou, Olympia Konstantakopoulou, Aglaia Katsiroumpa, Ioannis Moisoglou, Daphne Kaitelidou

**Affiliations:** Clinical Epidemiology Laboratory, Faculty of Nursing, National and Kapodistrian University of Athens, Athens,; Department of Radiology, P & A Kyriakou Children’s Hospital, Athens, Greece; University of Piraeus, Greece; Center for Health Services Management and Evaluation, Faculty of Nursing, National and Kapodistrian University of Athens, Athens, Greece; Faculty of Nursing, National and Kapodistrian University of Athens, Athens, Greece; Pulmonary Clinic, General Hospital of Lamia, Lamia, Greece; Center for Health Services Management and Evaluation, Faculty of Nursing, National and Kapodistrian University of Athens, Athens, Greece

**Keywords:** COVID-19, parents, vaccine uptake, children, Greece

## Abstract

**Background:**

As the COVID-19 pandemic continues to threaten public health, the vaccination of children against the disease appears to be a key factor to control the pandemic. Our aim was to investigate the prevalence of parents who have vaccinated their children against the COVID-19 and the factors influencing this decision.

**Materials and Methods:**

We conducted a web-based cross-sectional study in Greece during the first week of September 2021. The study questionnaire was distributed through social media and a convenience sample was obtained. Only parents with children aged 12–17 years old could participate in the study. We collected socio-demographic data of parents and we measured their attitudes towards vaccination and COVID-19 pandemic.

**Results:**

Study population included 656 parents. Regarding vaccination, 27.1% of parents had their children vaccinated against the COVID-19, while almost all children had a complete vaccination history (98.9%). The most important reasons for decline of COVID-19 vaccination were doubts about the safety and effectiveness of COVID-19 vaccines (45.3%) and fear of side effects (36.6%). Regarding the information about the COVID-19 vaccines, parents showed more trust in family doctors than in scientists and the government. We found that increased parents’ age, increased trust in COVID-19 vaccines, and positive attitude of parents towards vaccination had a positive effect on children’s vaccination.

**Conclusions:**

Understanding the factors influencing parents’ decision to vaccinate their children against the COVID-19 is crucial to increase the COVID-19 vaccination coverage rate. Implementation of public health policies is necessary to spread knowledge about COVID-19 vaccines and to regain vaccine confidence.

## Santrauka.

**Problema.:**

COVID-19 pandemijai toliau keliant grėsmę visuomenės sveikatai, atrodo, kad vaikų skiepijimas nuo šios ligos gali būti svarbiausias veiksnys siekiant suvaldyti pandemiją. Mūsų tikslas ištirti, kiek tėvų paskiepijo savo vaikus nuo COVID-19 ir kokie veiksniai lėmė šį sprendimą.

**Medžiaga ir metodai.:**

2021 m. rugsėjo pirmąją savaitę Graikijoje internetu atlikome vienmomentinį skerspjūvio tyrimą. Tyrimo anketa buvo platinama socialinėse medijose ir buvo gauta patogioji imtis. Tyrime galėjo dalyvauti tik 12–17 m. vaikus auginantys tėvai. Surinkome tėvų socialinius ir demografinius duomenis ir nustatėme jų požiūrį į skiepijimą ir COVID-19 pandemiją.

**Rezultatai.:**

Tyrime dalyvavo 656 tėvai. Skiepai – 27,1 % tėvų savo vaikus paskiepijo nuo COVID-19, o beveik visi vaikai buvo paskiepyti pagal visą skiepijimo schemą (98,9 %). Svarbiausios priežastys, dėl kurių buvo atsisakoma skiepyti nuo COVID-19, abejonės dėl skiepų saugumo ir veiksmingumo (45,3 %) ir šalutinio poveikio baimė (36,6 %). Informacija apie COVID-19 skiepus – tėvai labiau pasitikėjo šeimos gydytojais nei mokslininkais ir valdžia. Nustatėme, kad vaikus skiepyti buvo linkę vyresnio amžiaus tėvai, tėvai, pasitikintys COVID-19 skiepais, ir tėvai, teigiamai vertinantys skiepus.

**Išvados.:**

Norint didinti pasiskiepijusių nuo COVID-19 skaičių yra svarbu suprasti, kokie veiksniai turi įtakos tėvų sprendimui skiepyti savo vaikus nuo COVID-19. Yra būtina įgyvendinti visuomenės sveikatos politiką, kad būtų galima skleisti informaciją apie COVID-19 skiepus ir sugrąžinti pasitikėjimą skiepais.

## Introduction

The Coronavirus disease 2019 (COVID-19) pandemic continues to cause several problems at the individual, social and economic level, although from the beginning of 2021 there are several vaccines with proven safety and efficacy [1–4]. Successful management of the pandemic requires vaccination of the general population at a high rate, which may now be as high as 80% [5]. However, the COVID-19 vaccination rate exceeds 80% in only a few studies, and in some cases is extremely low, reaching as low as 28.6% [6]. Until now, COVID-19 vaccine uptake in most countries fall below estimates of the required threshold for herd immunity [7].

Unfortunately, in recent years there has been an increase in the general population’s negative attitude towards vaccination. Vaccine hesitancy refers to the reluctance of individuals to accept the vaccination offer. According to the World Health Organization, vaccine hesitancy is one of the top ten threats to public health [8]. Regarding children’s vaccination, the role of parents is crucial as they decide whether to vaccinate their children. In a recent systematic review, only 50–70% of Canadian 2-year-old children have received all of the recommended vaccines [9]. Parental vaccination decisions are complex and a variety of factors influence parents’ decision to vaccinate or not their children such as trust to healthcare providers, health perceptions and practices, healthcare system, attitudes toward vaccines, experiences, risks and effects of vaccination, religious issues, trust in science, information sources, emotions, etc. [9–12].

Unfortunately, COVID-19 cases in children continue to increase due mainly to the highly transmissible delta variant [13,14]. It is now clear that parents’ decision to vaccinate their children against the COVID-19 will be critical to control the pandemic. According to a meta-analysis, the overall willingness of parents to vaccinate their children against the COVID-19 is moderate (56.8%) and ranges from 29% to 72.7% [15]. Several factors affect parents’ intention to vaccinate their children against the COVID-19 such as socio-demographic variables, attitudes toward vaccines, knowledge, psychological factors, etc. [15]. However, to the best of our knowledge, no study to date has estimated the proportion of parents who have vaccinated their children against the COVID-19. Thus, the aim of our study was to investigate the prevalence of parents who have vaccinated their children against the COVID-19 and the factors influencing this decision.

## Materials and Methods

### Study design and participants

We conducted a web-based cross-sectional study in Greece during the first week of September 2021. From 15 July 2021 until the time of our study, the Greek government offered a free COVID-19 vaccine to all children aged 15–17 years old throughout the country. The vaccine was also offered to children aged 12–14 years old from 30 July 2021. The vaccination was voluntary and was carried out after the agreement of the parents or guardians. Data collection was carried out online using Google forms to create an anonymous version of the study questionnaire. The questionnaire was distributed through social media and a convenience sample was obtained. Only parents with children aged 12–17 years old could participate in the study. Information on the purpose and design of the study was provided at the beginning of the on-line questionnaire, and parents provided informed consent to participate anonymously in the study. Given that the percentage of children who are vaccinated against the COVID-19 is unknown, we considered a prevalence of 50% to estimate the largest sample size. Thus, considering the precision level as ±5% and the confidence level as 95%, a sample size of 385 parents was obtained. We subsequently decided to substantially increase the sample size to minimize random error. The Ethics Committee of Department of Nursing, National and Kapodistrian University of Athens approved the study protocol (reference number: 370, 02-09-2021).

### Questionnaire

We collected socio-demographic data of parents and we measured their attitudes towards vaccination and COVID-19 pandemic. Regarding socio-demographic data, we collected the following: gender, parents’ age, marital status, educational level, self-perceived financial status, self-perceived health status, parents’ chronic disease, parents’ previous COVID-19 diagnosis, family/friends with previous COVID-19 diagnosis, and living with elderly people or vulnerable groups during the COVID-19 pandemic. We used the five-point Likert scale from 0 (“very poor”), to 4 (“very good”) to measure financial status and self-perceived health status.

Regarding vaccination, we measured children’s and parents’ COVID-19 vaccination uptake, parents’ seasonal influenza vaccination in 2020 and children’s complete vaccination history. In all cases, we used “yes/no” answers. Also, we recorded the reasons why parents refuse to vaccinate their children.

Additionally, we recorded attitudes of parents towards vaccination and COVID-19 pandemic by measuring the following variables: self-perceived severity of COVID-19, self-perceived knowledge regarding COVID-19 and COVID-19 vaccines, concerns about the side effects of COVID-19 vaccination, trust in COVID-19 vaccines, the role of vaccination in ensuring public health, and trust in the government, scientists, and family doctors regarding the information about the COVID-19 vaccines. The above variables were measured on a scale from 0 to 10 with higher values indicating greater agreement.

### Statistical analysis

We used numbers (percentages) to present categorical variables and mean (standard deviation) to present continuous variables. The normality of the distribution of the continuous variables was assessed with the Kolmogorov–Smirnov test and normal Q-Q plots. The dependent variable was COVID-19 vaccine uptake in children. When parents vaccinated their children, the dependent variable was defined as 1. Socio-demographic variables and parents’ attitudes towards vaccination and COVID-19 pandemic were the independent variables. The relationship between the independent variables and the COVID-19 vaccine uptake in children was assessed with univariate and multivariate logistic regression analysis. Independent variables with p-values <0.20 in univariate logistic regression analysis were included in multivariate logistic regression analysis to eliminate confounding. We calculated unadjusted and adjusted odds ratios (OR), 95% confidence intervals (CI), and p-values. In multivariate logistic regression model, we applied the backward stepwise model. All tests of statistical significance were two-tailed. Statistical analysis was performed with the Statistical Package for Social Sciences software (IBM Corp. Released 2012. IBM SPSS Statistics for Windows, Version 21.0. Armonk, NY: IBM Corp.).

## Results

Study population included 656 parents. Table 1 presents socio-demographic data of parents. Mean age of parents was 45.5 years, while most of the participants were mothers (75.5%) and married (83.2%). Regarding educational level, 75.8% of parents had a University degree and 41.3% had a MSc/PhD degree. Regarding the COVID-19, 10.2% of parents were diagnosed with the disease and 58.8% of parents had family/friends with a previous COVID-19 diagnosis.

**Table 1.** Socio-demographic characteristics of parents.

Characteristics	N	%
Gender		
Mothers	495	75.5
Fathers	161	24.5
Parents’ age (years)^a^	45.7	7.7
Marital status		
Singles	34	5.2
Married	546	83.2
Divorced	71	10.8
Widowed	5	0.8
Educational level		
High school	159	24.2
University degree	497	75.8
MSc/PhD degree		
No	385	58.7
Yes	271	41.3
Self-perceived financial status		
Very poor	20	3.0
Poor	68	10.4
Moderate	353	53.8
Good	174	26.5
Very good	41	6.3
Self-perceived health status		
Very poor	4	0.6
Poor	26	4.0
Moderate	121	18.4
Good	305	46.5
Very good	200	30.5
Parents’ chronic disease		
No	508	77.4
Yes	148	22.6
Previous COVID-19 diagnosis		
No	589	89.8
Yes	67	10.2
Family/friends with previous COVID-19 diagnosis		
No	290	44.2
Yes	366	55.8
Living with elderly people or vulnerable groups during the COVID-19 pandemic		
No	453	69.1
Yes	203	30.9

a mean, standard deviation

Parents’ attitudes towards vaccination and COVID-19 pandemic are shown in Table 2. In the sample, 27.1% of parents had their children vaccinated against the COVID-19, while almost all children had a complete vaccination history (98.9%). The most important reasons for decline of COVID-19 vaccination were doubts about the safety and effectiveness of COVID-19 vaccines (45.3%), fear of side effects (36.6%), low self-perceived threat of COVID-19 for children (9.5%), and children that have already been diagnosed with COVID-19 (6.9%). Most parents were vaccinated against the COVID-19 (82.9%), while the respective percentage for seasonal influenza in 2020 was 54.3%. Parents’ self-perceived knowledge regarding COVID-19 and COVID-19 vaccines was very high, while trust in COVID-19 vaccines was high. Regarding the information about the COVID-19 vaccines, parents showed more trust in family doctors than in scientists and the government.

**Table 2.** Parents’ attitudes towards vaccination and COVID-19 pandemic.

	N	%
Children’s COVID-19 vaccination uptake		
No	478	72.9
Yes	178	27.1
Parents’ COVID-19 vaccination uptake		
No	112	17.1
Yes	544	82.9
Parents’ seasonal influenza vaccination in 2020		
No	300	45.7
Yes	356	54.3
Children’s complete vaccination history		
No	7	1.1
Yes	627	98.9
Reasons for decline of parents to vaccinate their children against the COVID-19		
I have doubts about the safety and effectiveness of COVID-19 vaccines	105	45.3
I am afraid of side effects of COVID-19 vaccines	85	36.6
I believe that even if my children get infected with COVID-19, nothing bad will happen to them	22	9.5
My children have already been diagnosed with COVID-19 and the vaccine will not be beneficial for them	16	6.9
Family physician does not allow me to vaccinate my children due to their medical condition	4	1.7
Self-perceived severity of COVID-19^a^	8.1	2.4
Self-perceived knowledge regarding COVID-19^a^	8.9	1.4
Self-perceived knowledge regarding COVID-19 vaccines^a^	8.6	1.8
Concerns about the side effects of COVID-19 vaccination^a^	6.0	3.2
Trust in COVID-19 vaccines^a^	6.9	3.2
COVID-19 vaccination promotes public health^a^	7.8	3.1
Vaccination is beneficial^a^	8.8	2.3
Trust in the government regarding the information about the COVID-19 vaccines^a^	5.2	3.5
Trust in scientists regarding the information about the COVID-19 vaccines^a^	7.1	3.2
Trust in family doctors regarding the information about the COVID-19 vaccines^a^	8.1	2.2

a mean, standard deviation

Table 3 presents univariate and multivariate logistic regression models. According to univariate analysis, increased parents’ age and higher educational level were associated with children’s COVID-19 vaccine uptake. Moreover, parents without a previous COVID-19 diagnosis and those who were vaccinated against the COVID-19 and seasonal influenza had a greater probability to vaccinate their children against the COVID-19. Increased self-perceived knowledge regarding COVID-19 and COVID-19 vaccines, self-perceived severity of COVID-19, and trust in COVID-19 vaccines, government, scientists and family doctors were related with children’s COVID-19 vaccine uptake. Additionally, positive attitude of parents towards vaccination was associated with increased vaccination of children. On the other hand, concerns about the side effects of COVID-19 vaccination reduced the likelihood of children being vaccinated.

Multivariate regression analysis identified that increased parents’ age (OR=1.07, 95% CI=1.04–1.10, p-value<0.001), increased trust in COVID-19 vaccines (OR=1.29, 95% CI=1.15–1.44, p-value<0.001) and positive attitude of parents towards vaccination (OR=1.42, 95% CI=1.09–1.86, p-value=0.001) had a positive effect on children’s vaccination.

**Table 3.** Univariate and multivariate logistic regression analysis with COVID-19 vaccine uptake in children as the dependent variable (reference: COVID-19 vaccine denial).

Variable	Unadjusted OR (95% CI)	P-value	Adjusted OR (95% CI)^a^	P-value
Gender (mothers vs. fathers)	1.03 (0.69 – 1.54)	0.89	NS	
Parents’ age (years)	1.08 (1.05 – 1.10)	<0.001	1.07 (1.04 – 1.10)	<0.001
Marital status (singles/widowed/divorced vs. married)	1.01 (0.64 – 1.59)	0.97	NS	
Educational level (University degree vs. High school)	1.91 (1.23 – 2.99)	0.004	NS	
MSc/PhD degree (yes vs. no)	1.35 (0.95 – 1.91)	0.09	NS	
Self-perceived financial status			NS	
Good/very good	1.21 (0.69 – 2.09)	0.50		
Moderate	0.87 (0.51 – 1.48)	0.61		
Very poor/poor	1 (reference)			
Self-perceived health status			NS	
Good/very good	1.57 (0.63 – 3.91)	0.34		
Moderate	1.32 (0.49 – 3.53)	0.58		
Very poor/poor	1 (reference)			
Parents’ chronic disease (yes vs. no)	1.08 (0.72 – 1.63)	0.70	NS	
Previous parents’ COVID-19 diagnosis (no vs. yes)	2.59 (1.26 – 5.35)	0.01	NS	
Family/friends with COVID-19 disease (no vs. yes)	1.29 (0.92 – 1.83)	0.14	NS	
Living with elderly people or vulnerable groups during the COVID-19 pandemic (yes vs. no)	1.28 (0.89 – 1.84)	0.19	NS	
Parents’ COVID-19 vaccination uptake (yes vs. no)	17.23 (5.39 – 55.01)	<0.001	NS	
Parents’ seasonal influenza vaccination in 2020 (yes vs. no)	1.58 (1.11 – 2.24)	0.01	NS	
Self-perceived severity of COVID-19	1.42 (1.27 – 1.59)	<0.001	NS	
Self-perceived knowledge regarding COVID-19	1.31 (1.12 – 1.52)	0.001	NS	
Self-perceived knowledge regarding COVID-19 vaccines	1.36 (1.19 – 1.55)	<0.001	NS	
Concerns about the side effects of COVID-19 vaccination	0.84 (0.79 – 0.89)	<0.001	NS	
Trust in COVID-19 vaccines	1.43 (1.29 – 1.57)	<0.001	1.29 (1.15 – 1.44)	<0.001
COVID-19 vaccination promotes public health	1.55 (1.36 0 1.76)	<0.001	NS	
Vaccination is beneficial	1.93 (1.53 – 2.44)	<0.001	1.42 (1.09 – 1.86)	0.01
Trust in the government regarding the information about the COVID-19 vaccines	1.18 (1.11 – 1.24)	<0.001	NS	
Trust in scientists regarding the information about the COVID-19 vaccines	1.28 (1.19 – 1.38)	<0.001	NS	
Trust in family doctors regarding the information about the COVID-19 vaccines	1.26 (1.14 – 1.40)	<0.001	NS	

An odds ratio <1 indicates a negative association, while an odds ratio >1 indicates a positive association.

CI: confidence interval; NS: not selected by the backward elimination procedure in the multivariable logistic regression analysis with a significance level set at 0.05; OR: odds ratio

a R^2^ for the final multivariate model was 27%

## Discussion

To our knowledge, this is the first study that estimates COVID-19 vaccine uptake among children and investigates the factors that lead parents to vaccinate their children against the COVID-19. In a sample of parents in Greece, we found that 27.1% of them had already vaccinated their children aged 12–17 years old. This proportion seems low, but the absence of similar studies does not allow us to make direct comparisons with other countries and draw safer conclusions. Furthermore, our study was conducted just one month after the Greek government approved COVID-19 vaccines for children. As evidence from studies increases, it is quite likely that more parents will be persuaded to vaccinate their children against the COVID-19. Early results of randomized controlled trials on the safety and efficacy of COVID-19 vaccines in children are encouraging [16,17], but they are relatively few and it is therefore reasonable for parents to approach COVID-19 vaccines with thoughtfulness and disinclination. Increased parental knowledge about vaccination is a key factor in reducing parents’ concerns and increasing parents’ positive attitude toward vaccination [18–20].

Consistent with prior studies in adults [21–24], the most important reasons for decline of COVID-19 vaccination were concerns about the safety and effectiveness of COVID-19 vaccines and fear of side effects. In the case of children, the issue is more complicated, as parents feel responsible for their children’s health and are reluctant to vaccinate their children with a novel vaccine for which there is not yet enough data from studies. It is essential to increase parental knowledge, as this can significantly reduce parents’ vaccine hesitancy [25,26]. Parents, even those who are opposed to vaccination, seek as much and more valid scientific information as possible, so that they can make their final decision [27].

An interesting finding of our study is the fact that most parents had been vaccinated against the COVID-19 (82.9%) and more than half had been vaccinated against influenza (54.3%). This finding is extremely encouraging, as it indicates a positive attitude of parents towards vaccination at a time when the COVID-19 vaccination rate among adults is low [22,28–30]. Moreover, we found that positive attitude of parents towards vaccination had a positive effect on children’s vaccination. Literature confirms this finding since the successful implementation of childhood vaccination programmes depends to a large extent on the positive attitude of parents [19,31,32]. Awareness campaigns and distribution of leaflets about vaccination have positive effects and are critical in changing the attitudes of parents toward vaccinating their children [33,34]. Also, individual stories from COVID-19 patients and positive experiences of vaccinated people could promote positive attitude of parents towards vaccination [35].

Our multivariate regression model revealed that parents who had more trust in COVID-19 vaccines were more likely to vaccinate their children against the disease. Several studies have already shown that trust in COVID-19 vaccines is related with parents’ willingness to vaccinate their children against the COVID-19 [36–38]. Vaccine hesitancy today is a major public health issue and is mainly due to a lack of trust. It is essential to develop a fruitful dialogue between citizens, scientists, policymakers and the government, so as to create a relationship of trust and to implement up-to-date health policies on COVID-19 vaccination [35]. Furthermore, the role of the mass media is particularly important, as the spread of fake news reduces both confidence in COVID-19 vaccines and the intention to vaccinate [39]. On-line COVID-19 information from websites is often of low quality and should therefore be systematically monitored by impartial governmental bodies [40–42]. We emphasize the crucial role of family doctors in informing parents as we found that parents trust family doctors more and scientists and government less. For this reason, medical associations should continuously inform and train family doctors on the appropriate way to approach and inform parents on controversial issues such as vaccination.

Our findings should be interpreted in the context of several limitations. Given the convenience sample, the on-line data collection, and the unknown response rate, the extent to which our findings can be generalized to other parents is unknown. For instance, parents with limited internet access were less likely to participate in our study. There may have been an information bias in our study since vaccine uptake was self-reported and some parents may have falsely reported that they vaccinated their children. However, the fact that the questionnaire was completed anonymously may have reduced this bias. Moreover, we have explored several factors that may influence parents’ decision to vaccinate their children, but clearly there are other factors that can be studied such as psychological factors, mass media variables, impact of fake news, etc. Finally, our sample included mainly mothers with a high level of education. Thus, further studies with representative samples are critically needed to draw safer conclusions.

## Conclusions

We provide an early assessment of children’s COVID-19 vaccination status in Greece. Understanding the factors influencing parents’ decision to vaccinate their children against the COVID-19 is crucial to increase the COVID-19 vaccination coverage rate. Future research is needed in this area, as the factors influencing the parents’ decision are miscellaneous. In addition, evidence from studies regarding COVID-19 vaccines is constantly increasing which may influence parents’ attitudes. Mandatory vaccination does not seem to be the most appropriate approach as it generates strong reactions and disagreements. For this reason, the establishment of mutual trust between parents and the scientific community is an essential prerequisite for making a rational decision regarding COVID-19 vaccination in children. Implementation of public health policies is necessary to spread knowledge about COVID-19 vaccines and to regain vaccine confidence.
